# Supplementary material for: CC5 and CC8, Two Disintegrin Isoforms from Cerastes cerastes Snake Venom Decreased Inflammation Response In Vitro and In Vivo
Source: Int J Mol Sci. 2023 Aug 4;24(15):12427. doi: 10.3390/ijms241512427 (PMC10418880; doi:10.3390/ijms241512427)
Supplement: Supplementary file 1 [file ijms-24-12427-s001.zip › ijms-2497082-supplementary.pdf]

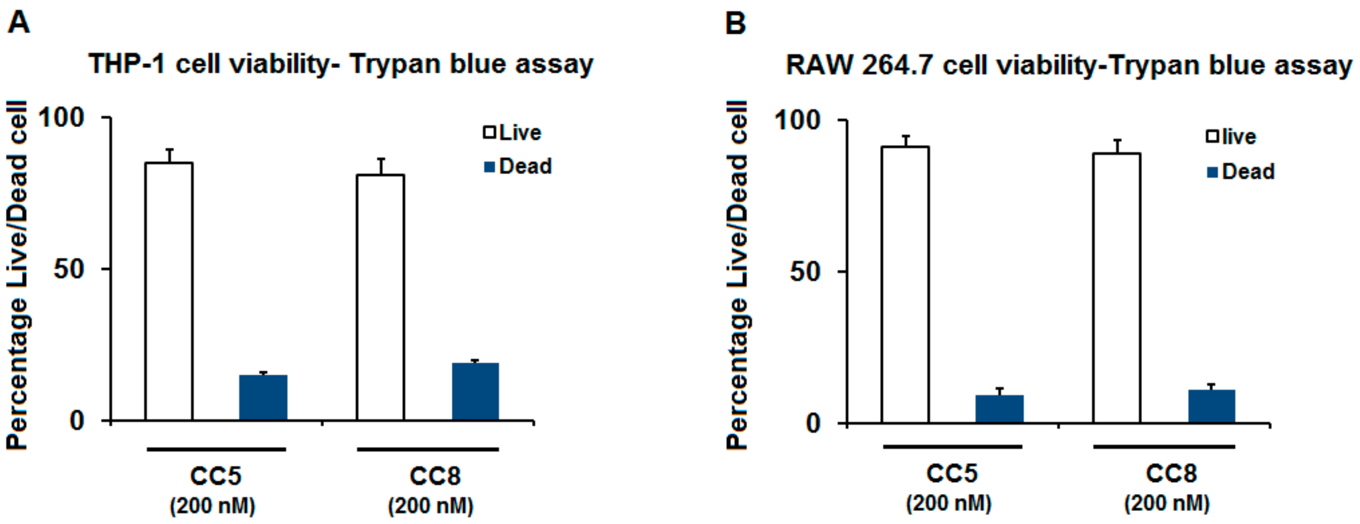


**Figure S1:** Trypan blue viability assay showing the percentage of live and dead cells treated with 200 nM of CC5 and CC8 in THP-1 cells (A) and RAW264.7 Cells (B) at 24 h. All values are expressed as mean ± standard error of the mean (SEM) from three different samples.
